# Accuracy of laboratory tests drawn by pull-push method from central venous catheterization after routine flushing with 10 ml normal saline in patients with sepsis at the emergency department

**DOI:** 10.1016/j.heliyon.2021.e07355

**Published:** 2021-06-23

**Authors:** Thanin Lokeskrawee, Sombat Muengtaweepongsa, Jayanton Patumanond, Chutinun Sawaengrat

**Affiliations:** a Department of Emergency Medicine, Lampang Hospital, Muang District, Lampang, 52000, Thailand; b Center of Excellence in Stroke, Department of Internal Medicine, Thammasat University, Pathum Thani 12120, Thailand; c Center for Clinical Epidemiology and Clinical Statistics, Faculty of Medicine, Chiang Mai University, Chiang Mai, 50200, Thailand

**Keywords:** Accuracy, Laboratory tests, Blood specimen collection, Catheterization, Central venous, Pull-push method

## Abstract

Central venous catheterization (CVC) remains a common practice in the emergency setting. Routine flushing 10–20 ml of normal saline to maintain the patency of CVC could affect the accuracy of laboratory tests. Typically, physicians require peripheral vein phlebotomy when more blood sampling is needed. One alternative method, the Pull-push method, could avoid the trauma associated with venipuncture and unnecessary peripheral vein phlebotomy. However, there has been no recent study analyzing the accuracy of blood sampling using this technique. We evaluate laboratory tests' accuracy between blood samples drawn by the Pull-push method from CVC after routine flushing with 10 ml of normal saline versus control. We conducted a diagnostic accuracy study from May to September 2019. After exclusion, 72 patients were eligible for analysis. Promptly after central venous catheterization, we drew blood samples, stored them in blood collecting tubes, and labeled them for the gold standard group. We flushed with 10 ml of normal saline before blood sampling using the Pull-push method's completed three times; then, we drew blood samples again, labeled Pull-push group. We compared the laboratory results between two groups by paired t-test. The accuracies were analyzed based on an allowable error by Clinical Laboratory Improvement Amendments (CLIA) and presented by a modified Bland-Altman plot. The 72 patients were primarily male (n = 47, 65.3%), had a mean age 60.1 ± 14.0 years, and were diagnosed with sepsis (n = 4, 5.6%) or septic shock (n = 65, 90.3%). For almost all the laboratory values, including hemoglobin, hematocrit, white blood cell count, platelet count, blood urea nitrogen, creatinine, sodium, potassium, chloride, bicarbonate, prothrombin time, international normalized ratio, and blood sugar, the accuracy was more than 90% (92.8–98.6%), except aPTT (85.5%) and aPTT ratio (86.7%). Laboratory tests drawn by the Pull-push method could replace peripheral vein phlebotomy to avoid the trauma associated with venipuncture and infection risk.

## Teaser

Accuracy; Laboratory tests; Blood specimen collection; Catheterization; Central cenous; Pull-push method.

## 1 Introduction

Central venous catheterization remains a standard procedure in the emergency department. After the procedure, it is mandatory to flush 10–20 ml of the saline to prevent clot formation in central venous catheters (CVC). However, physicians usually request other blood samples in critically ill patients. Patients need to be re-drawn from peripheral blood, which is sometimes tricky owing to collapsed arteries due to shock. In addition, patients may get the trauma associated with venipuncture from multiple punctures, and their veins may become multiple punctures leading to local inflammatory complications [1].

From the above problem, therefore, many previous studies have been conducted to reduce unnecessary blood venipuncture. The methods used by previous studies in patients with a peripherally inserted central catheter (PICC), tunneled catheter, or permanent catheter were summarized as follows: 1) Discard method [1, 2, 3] by draw blood (waste volume): the volume depends on the dead space of each catheter. After waste volume discard, physicians then collect blood samples for a laboratory test. However, this method may affect the patient's hemodynamic, especially patients with anemia due to blood loss. 2) Reinfusion method [4] by drawing waste volume and holding blood samples for testing: the waste volume is returned to the patient. The limitation is that the clotting-initiated blood may be returned to the patient [5] and may increase the chance of infection [6]. 3) Push-Pull method [7]: the method is to push-pull blood in and out approximately three times in pediatric patients with permanent catheters for chemotherapy. The study said that the laboratory results were accurate compared with the Discard method to reduce blood loss. Moreover, the Push-Pull method can maintain the circulatory system's stability and reduce the problem of infection. However, no such studies have been conducted in the emergency department for CVC patients. Therefore, we investigate the accuracy of laboratory results by the Pull-push method compared with control in CVC patients.

## 2 Materials & methods

### 2.1 Patients and setting

We conducted a diagnostic accuracy study at the Department of Emergency Medicine, Lampang Hospital, Thailand, from May 2019 to September 2019. Patients who were older than 18 years of age with indication and no contraindications for CVC were enrolled. We obtained CVC via right internal jugular catheterization. All participants signed informed consent before enrollment. We excluded: 1) patients who refused to participate, 2) pregnant women, 3) technical errors such as blood clots or hemolysis, and 4) patients with immediate complications such as pneumothorax.

The surgical equipment used in CVC placement was of identical make and model, with a 16/16-gauge double lumen, locking both lumens to prevent air from entering. Furthermore, for blood samples' accuracy in this study's gold standard group, normal saline was not pre-cast in the CVC line. However, all patients were in Trendelenburg's position to prevent air embolism [8].

We cleaned the skin with the same 2% chlorhexidine-gluconate in 70% alcohol solution without povidone-iodine in every case before the procedure. This is because studies have shown that povidone-iodine may distort laboratory results [9], and 2% chlorhexidine-gluconate in 70% alcohol solution should be enough to reduce infection [10].

After successful CVC insertion, we collected 12 ml of blood samples in the control group. The samples were then placed in blood collecting tubes, sorted according to Clinical Laboratory Improvement Amendments (CLIA) guidelines [11] labeled as a gold standard group. We flushed the line with 10 ml of normal saline and used an empty syringe (10 ml empty syringe) to perform three pull-push sessions. The pull-push session included slowly pulling 10 ml blood from the line for 5 s duration, gently pushing the 10 ml of blood back into the line for 5 s, and then waiting for 5 s before beginning the next pull-push session. Next, we used another empty syringe to collect 12 ml blood samples and labeled them as a Pull-push group. The time interval for the logistic process was similar in both groups. Other external factors were kept similar between groups.

The study was approved by the Human Research Ethics Committee, Lampang Hospital.

Definition [12]

1. Percentage error (% error): the error of the laboratory tests from the Pull-push group was compared with the gold standard using the equation: (laboratory test results from the Pull-push group — the gold standard) x 100/laboratory test results from the gold standard.
2. Allowable error: the acceptable percentage errors as defined by the CLIA [13, 14]:
  Prothrombin time (PT): ±15%;
  International normalized ratio (INR): ±15%;
  Activated partial thromboplastin time (aPTT): ±15%;
  aPTT ratio: ±15%;
  Hemoglobin (Hb): ±7%;
  Hematocrit (Hct): ±6%;
  White blood cell (WBC): ±15%;
  Platelet count: ±25%;
  Blood urea nitrogen (BUN): ±9%;
  Creatinine (Cr): ±15%.
  Sodium (Na): ±4 mmol/L;
  Potassium (K): ±0.5 mmol/L;
  Chloride (Cl): ±5%;
  Bicarbonate (HCO3): ±20%; and.
  Blood sugar (BS): ±10%.
3. Accuracy (100%-Percentage error): The percentage of allowable error for each laboratory test. Accuracy ≥90% is considered highly accurate.

### 2.2 Study size estimation

Based on the pilot study, the mean hemoglobin of the Pull-push group and gold standard group was 8.8 ± 2.7 vs. 9.7 ± 2.7 gm/dL. To obtain the power of 80%, an alpha error of 5%, with a two-sided test, this study required at least 72 patients to validate the various laboratory tests.

### 2.3 Safety protocols

1. The operative doctor must be a senior emergency medicine resident under the supervision of a medical instructor
2. All patients needed a pulse oximeter, electrocardiogram, and blood pressure monitoring for safety.
3. Provision of appropriate care in case of complications. Patients and relatives would get notified and excluded from the study.
4. We monitored the complications on days 1, 2, and 7 after the procedure.

### 2.4 Statistical analysis

Baseline characteristics were presented by number (percentage) for categorical data and by mean ± SD for numerical data. The mean value of laboratory tests between the Pull-push group and the gold standard group were compared using paired t-tests, with laboratory tests having a p-value of >0.05 being interpreted as insignificant. Mean difference and percentage error were also analyzed and presented using modified Bland-Altman plots. We calculated the accuracy for each laboratory test according to the CLIA standard.

## 3 Results

**Figure 1:**
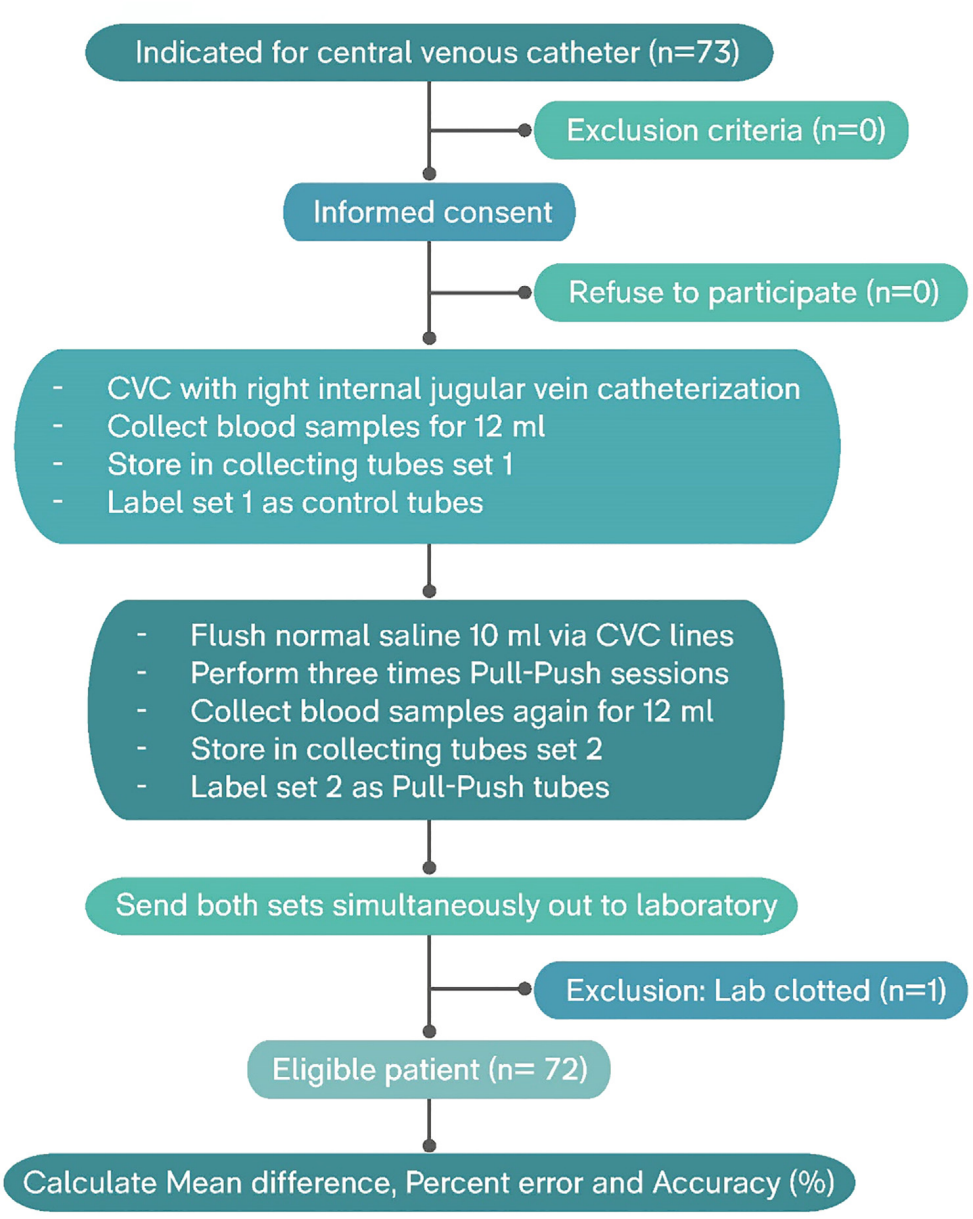
Study flow.

From May to September 2019, 73 patients met the study criteria. After excluding one sample with clotting blood (n = 1, 1.4%), 72 remaining patients were eligible for analysis (Figure 1).

Most of the patients were male, mean age 60.1 ± 14.0 years, almost all were diagnosed with sepsis and septic shock (Table 1).

**Table 1:** Demographic data.

Variable	Number N = 72	Percent
Gender		
Male	47	65.3
Female	25	34.7
Age (year) (Mean, ± SD)	60.1 ± 14.0	
Underlying:		
Diabetes mellitus	17	23.6
Hypertension	23	31.9
Hyperlipidemia	8	11.1
Kidney disease	12	16.7
Current antithrombotic:		
Antiplatelets	13	18.1
Anticoagulants	0	0
Both	0	0
None	59	81.9
Diagnosis:		
Sepsis	4	5.6
Septic shock	65	90.3
Other	3	4.1
Complications:		
Pneumothorax	0	0
Infection	0	0

### 3.1 Mean values, mean differences, and percentage errors

The mean differences of laboratory tests varied from -0.2 ± 1.0 to 1.1 ± 2.6 units. The mean of laboratory results by the Pull-push method and gold standard group were not different except for coagulogram (PT, INR, aPTT, aPTT ratio.) (Table 2).

**Table 2:** Mean, Percent error and Accuracy (%) of all laboratory results in each group.

Laboratory	Mean of the reference value (gold standard)	Mean of Pull-Push method	p-value	Mean difference Pull-Push method vs Gold standard	Allowable error (%)	Accuracy (%)
Hb (gm/dL)	9.3 ± 2.7	9.3 ± 2.7	0.175	-0.0 ± 0.3	±7%	92.8
Hct (vol%)	28.5 ± 8.0	28.3 ± 7.9	0.059	-0.2 ± 1.0	±6%	94.2
WBC (x10^3^cell/mm^3^)	15.9 ± 12.0	15.8 ± 12.1	0.169	-0.1 ± 0.6	±15%	97.1
Platelet (x10^3^cell/mm^3^)	182.9 ± 124.9	182.9 ± 124.9	0.961	0.1 ± 12.3	±25%	94.2
BUN (mg/dL)	43.0 ± 38.8	42.9 ± 39.1	0.587	-0.1 ± 1.1	±9%	98.6
Cr (mg/dL)	2.8 ± 2.5	2.8 ± 2.5	0.919	0.0 ± 0.1	±15%	97.1
Na (mmol/L)	135.7 ± 6.7	135.9 ± 6.6	0.141	0.2 ± 1.2	±4 mmol/L	95.7
Cl (mmol/L)	103.2 ± 7.7	103.4 ± 7.8	0.052	0.2 ± 0.8	±5%	95.7
K (mmol/L)	3.7 ± 0.9	3.7 ± 0.9	1.000	0.0 ± 0.1	±0.5 mmol/L	95.8
HCO_3_ (mmol/L)	18.5 ± 5.6	18.7 ± 5.7	0.206	0.2 ± 1.1	±20%	94.2
PT (sec)	17.5 ± 5.9	17.7 ± 5.9	0.009	0.1 ± 0.4	±15%	97.1
INR	1.5 ± 0.4	1.5 ± 0.4	0.013	0.0 ± 0.0	±15%	98.6
aPTT (sec)	32.9 ± 13.9	34.1 ± 14.2	0.001	1.1 ± 2.6	±15%	85.5
aPTT ratio	1.3 ± 0.5	1.3 ± 0.6	0.001	0.0 ± 0.1	±15%	86.7
Blood sugar (mg/dL)	182.1 ± 205.4	182.0 ± 205.1	0.841	-0.1 ± 4.3	±10%	94.2

**Figure 2:**
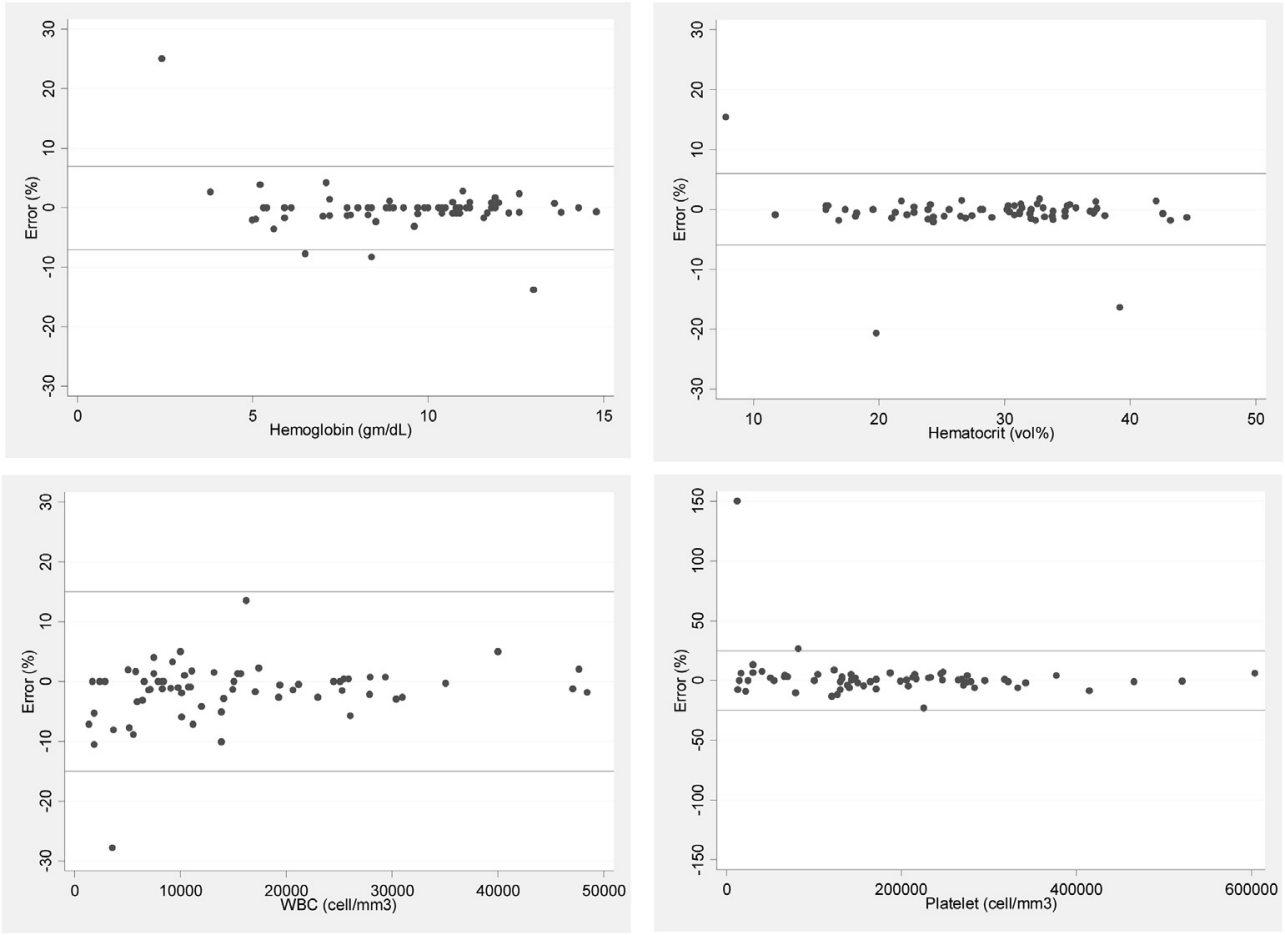
Modified Bland-Altman plots of complete blood count (hemoglobin, hematocrit, white blood cell count [WBC], and platelet count).

**Figure 3:**
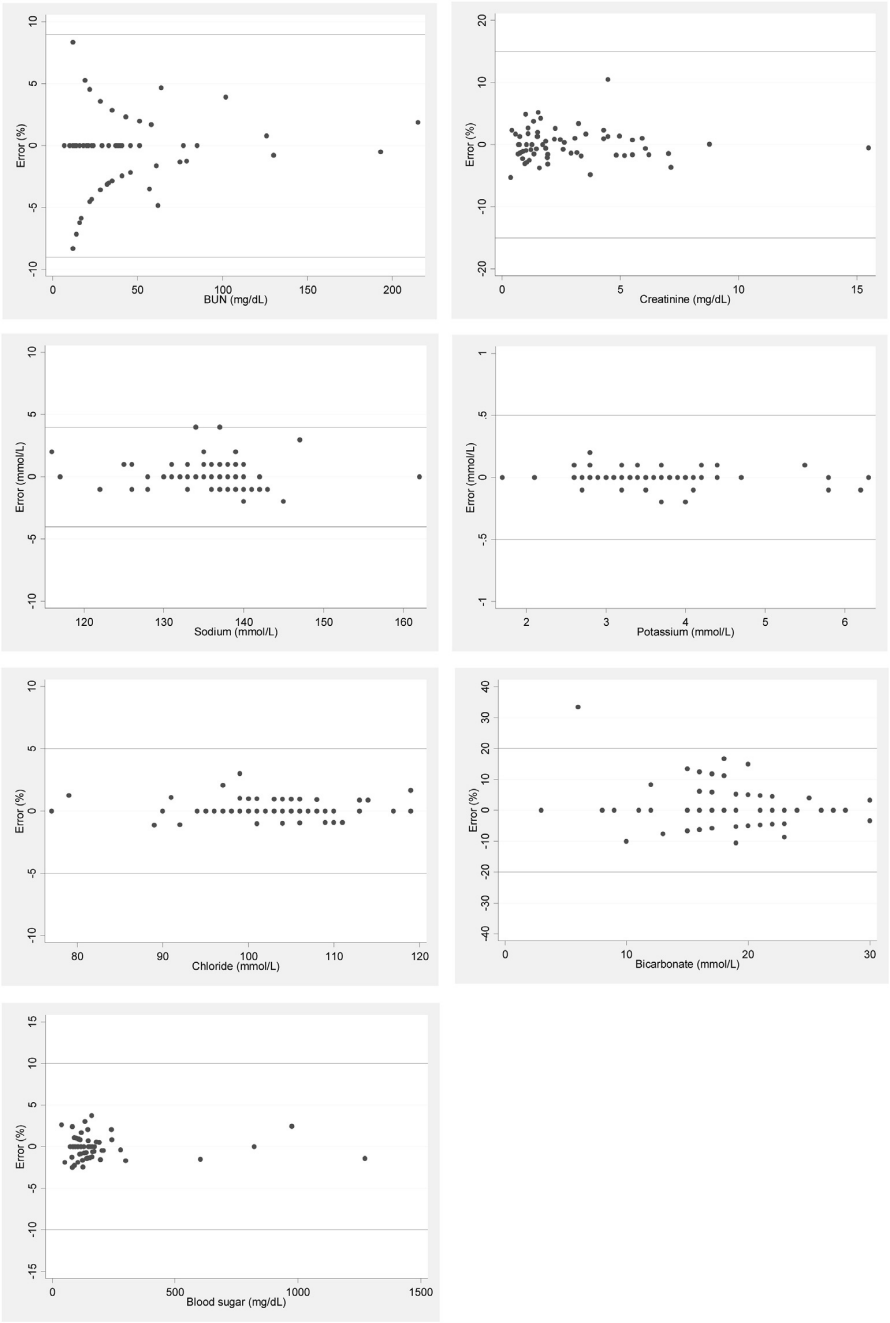
Modified Bland-Altman plots of serum chemistry (blood urea nitrogen, [BUN], and creatinine), serum electrolytes (sodium, potassium, chloride, bicarbonate, and blood sugar).

**Figure 4:**
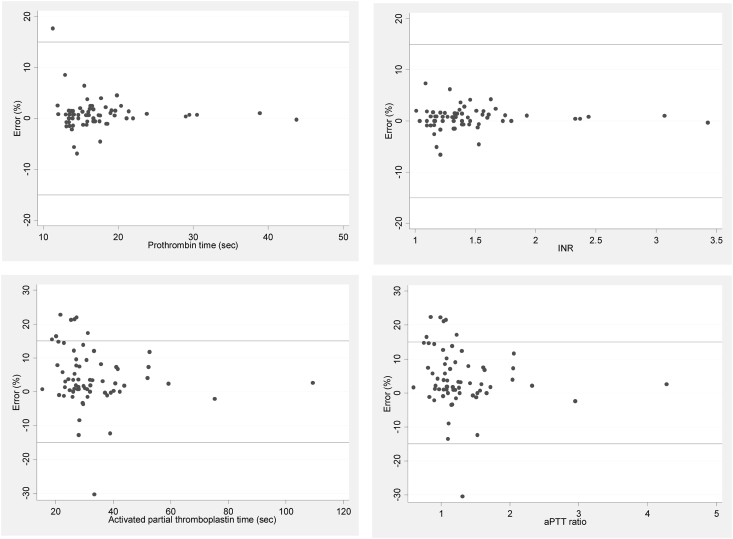
Modified Bland-Altman plots of coagulogram (prothrombin time, the international normalized ratio [INR], activated partial thromboplastin time [aPTT], and aPTT ratio).

The percentage errors of laboratory tests were presented by modified Bland-Altman plots, complete blood count (Figure 2), electrolytes, blood sugar (Figure 3), and coagulogram (Figure 4), which were all in the allowable error range except aPTT and aPTT ratio.

### 3.2 The accuracy

When analyzing accuracy based on CLIA's allowable error criteria [13, 14], we found that laboratory results using the Pull-push method were highly accurate (92.8%–98.6%) except aPTT (85.5%) and aPTT ratio (86.7%) (Table 2).

## 4 Discussion

For patients undergoing CVC with the right internal jugular vein after flush normal saline 10 ml in routine practice, almost all laboratory results show very high accuracy with three Pull-push methods (92.8%–98.6%). The laboratory with high accuracy results includes Hb, Hct, WBC, platelet count, BUN, Cr, Na, Cl, K, HCO3, PT, INR, and blood sugar. The errors seem to be high in aPTT and its ratio. However, those errors are still within the allowable range. According to CLIA standards [13, 14], this study's results benefit sepsis patients at the Emergency Department. Therefore, physicians may avoid unnecessary blood drawing from peripheral lines. On average, physicians need to draw blood samples from the peripheral lines 6–8 times in a single patient. Moreover, patients in shock with flattened veins pose difficulty in drawing blood.

Physicians previously recognized the Discard method [1, 2, 3] as having the same accuracy as standard. However, to avoid unnecessary blood loss (waste volume) from the Discard method, physicians usually re-infuse the waste blood back into the central lines [4]. This re-infusion may increase the risk of infection and may mistakenly return blood clots to the patient. In our study, the Pull-push method [7], using an empty syringe of 10 ml to pull the blood in and out three times, is safe for most patients. In addition, the patient's vital signs remain stable with the Pull-push method.

In contrast to our study, the patients in other previous Pull-push methods are mostly cancer patients. They all aimed to study coagulation tests' accuracy [1, 2, 3] to evaluate safety before invasive procedures, monitor and follow up treatment. Unfortunately, there are lacking studies on critically ill patients in the emergency department or intensive care unit [15].

From systematic reviews of 11 studies [16], they find that sample sizes in each study were too small (N range from 12-53) (72 sample sizes in this study). Pearson correlations, or mean difference comparisons, may have statistical differences but cannot be interpreted as clinical judgment, making them difficult to apply in actual practice. In our study, allowable errors are corrected mainly by reference to CLIA [13, 14].

Serum potassium is the most vulnerable one. CLIA uses a different error to justify serum potassium accuracy instead of a percent error as usual. Therefore, serum potassium has a very narrow acceptable difference. The serum potassium difference needs to be not greater than ±0.5 mmol/L to become acceptable. Our study shows that serum potassium with the Pull-push method is still accurate at 95.8%. This high accuracy in such a vulnerable laboratory gives confidentiality to the Pull-push method in other laboratory subtypes. However, high accuracy in serum potassium does not essentially verify the pull-push method's accuracy in itself.

The aPTT and its ratio have a low accuracy of only 85.5% and 86.7%, respectively. Compared to other coagulograms such as PT and INR, the accuracy is as high as 98.1% and 97.1%, respectively. Fortunately, PT and INR are more valuable than aPTT in an emergency setting, such as stroke, sepsis, and accident. Therefore, the low accuracy of aPTT and its ratio should not impact much to routine clinical practice. However, further study may help to answer whether this low accuracy may prevent using the Pull-push method in aPTT.

Our study highlights the use of control group blood samples drawn from the same CVC line before flushing normal saline 10 ml, then the Pull-push method, consistent with routine clinical practice. Our study design has an advantage over other studies using blood samples from peripheral phlebotomy as a control, where the values are significantly different [2, 3, 15, 17].

## 5 Conclusions

Laboratory tests drawn by the CVC line's Pull-push method could replace peripheral vein phlebotomy to reduce too much patients' pain and risk of infection.

## Declarations

### Author contribution statement

Thanin Lokeskrawee: Conceived and designed the experiments; Performed the experiments; Analyzed and interpreted the data; Contributed reagents, materials, analysis tools or data; Wrote the paper.

Sombat Muengtaweepongsa: Conceived and designed the experiments; Contributed reagents, materials, analysis tools or data; Wrote the paper.

Jayanton Patumanond: Conceived and designed the experiments; Analyzed and interpreted the data; Contributed reagents, materials, analysis tools or data.

Chutinun Sawaengrat: Performed the experiments.

### Funding statement

This research did not receive any specific grant from funding agencies in the public, commercial, or not-for-profit sectors.

### Data availability statement

Data will be made available on request.

### Declaration of interests statement

The authors declare no conflict of interest.

### Additional information

No additional information is available for this paper.
